# ‘*Ca*. Liberibacter asiaticus’ Proteins Orthologous with *pSymA*-Encoded Proteins of *Sinorhizobium meliloti*: Hypothetical Roles in Plant Host Interaction

**DOI:** 10.1371/journal.pone.0038725

**Published:** 2012-06-25

**Authors:** L. David Kuykendall, Jonathan Y. Shao, John S. Hartung

**Affiliations:** United States Department of Agriculture, Agricultural Research Service, Molecular Plant Pathology Laboratory, Beltsville, Maryland, United States of America; Instituto Butantan, Brazil

## Abstract

*Sinorhizobium meliloti* strain 1021, a nitrogen-fixing, root-nodulating bacterial microsymbiont of alfalfa, has a 3.5 Mbp circular chromosome and two megaplasmids including 1.3 Mbp *pSymA* carrying nonessential ‘accessory’ genes for nitrogen fixation (*nif)*, nodulation and host specificity (*nod*). A related bacterium, psyllid-vectored ‘*Ca*. Liberibacter asiaticus,’ is an obligate phytopathogen with a reduced genome that was previously analyzed for genes orthologous to genes on the *S. meliloti* circular chromosome. In general, proteins encoded by pSymA genes are more similar in sequence alignment to those encoded by *S. meliloti* chromosomal orthologs than to orthologous proteins encoded by genes carried on the ‘Ca. Liberibacter asiaticus’ genome. Only two ‘*Ca*. Liberibacter asiaticus’ proteins were identified as having orthologous proteins encoded on *pSymA* but not also encoded on the chromosome of *S. meliloti.* These two orthologous gene pairs encode a Na^+^/K+ antiporter (shared with intracellular pathogens of the family *Bartonellacea*) and a Co++, Zn++ and Cd++ cation efflux protein that is shared with the phytopathogen *Agrobacterium*. Another shared protein, a redox-regulated K+ efflux pump may regulate cytoplasmic pH and homeostasis. The pSymA and ‘*Ca*. Liberibacter asiaticus’ orthologs of the latter protein are more highly similar in amino acid alignment compared with the alignment of the pSymA-encoded protein with its *S. meliloti* chromosomal homolog. About 182 *pSymA* encoded proteins have sequence similarity (≤E-10) with ‘*Ca*. Liberibacter asiaticus’ proteins, often present as multiple orthologs of single ‘*Ca*. Liberibacter asiaticus’ proteins. These proteins are involved with amino acid uptake, cell surface structure, chaperonins, electron transport, export of bioactive molecules, cellular homeostasis, regulation of gene expression, signal transduction and synthesis of amino acids and metabolic cofactors. The presence of multiple orthologs defies mutational analysis and is consistent with the hypothesis that these proteins may be of particular importance in host/microbe interaction and their duplication likely facilitates their ongoing evolution.

## Introduction

Within the order Rhizobiales of the class alpha-Proteobacteria, *Sinorhizobium meliloti* is a member of a diverse bacterial family, *Rhizobiaceae* [1]–[3]. Other genera contained within the *Rhizobiaceae* and indeed the *Rhizobium* type genus [3] include *Agrobacterium* and *Rhizobium* [4]. A growing number of species of the family *Rhizobiaceae* and of the order *Rhizobiales* are becoming recognized based on 16 S RNA gene sequence data. Many species interact with plants and other eukaryotes to produce important agricultural, economic and/or environmental consequences. ‘*Ca.* Liberibacter asiaticus’ is recognized as the causal agent of huanglongbing (HLB), also known as citrus greening disease. This phytopathogen was introduced into the New World from Asia [5], [6], and is transmitted by the citrus psyllid, *Diaphorina citri* [7]. The full genomic sequence of ‘*Ca*. Liberibacter asiaticus’ was determined by deep sequencing of total DNA obtained from a single citrus psyllid which contained more than 10^8^ cells of ‘*Ca*. Liberibacter asiaticus.’ Phylogenetic analysis indicated that ‘*Ca*. Liberibacter asiaticus’ and *S. meliloti* are close bacterial relatives [2]. ‘*Ca.* Liberibacter asiaticus’ has a single small chromosome of 1.23 Mbps with 36.5% GC content compared to the chromosome of *S. meliloti* which is 3.65 Mbps and 62.7% GC [2], [8]. ‘*Ca*. Liberibacter asiaticus’ is not available in culture but can colonize intracellularly and move systemically in the phloem vessels of citrus trees [9], [10] following introduction by the citrus psyllid, *D. citri*. ‘*Ca*. Liberibacter asiaticus’ also systemically and intracellularly colonizes citrus psyllid salivary glands [11]. The genome of ‘*Ca*. Liberibacter asiaticus’ has many adaptations to facilitate an intracellular lifestyle in both plant and insect cells [2], [12].

*S. meliloti* 1021, free living in the soil, also lives in specialized root nodules in symbiosis with alfalfa, *Medicago sativa* L., where *S. meliloti* reduces atmospheric nitrogen and thereby confers plant nitrogen sufficiency. *S. meliloti* 1021 has a composite genome comprised of the chromosome and two megaplasmids, *pSymA* and *pSymB,* of 1.3 Mbps and 1.7 Mbps [8]. Megaplasmids *pSymA* and *pSymB* encode 1293 and 1570 protein-coding genes, respectively [8], [13].

Predicted gene products of all open-reading frames (ORFs) in the genome of ‘*Ca.* Liberibacter asiaticus’ were compared by protein BLAST against all of the predicted gene products encoded by chromosomal genes of *S. meliloti* 1021 [2]. However, predicted gene products of all ORFs encoded on the plant symbiosis-controlling *S. meliloti* megaplasmid *pSymA* had not been previously subjected to protein BLAST compared with the predicted gene products encoded in the ‘*Ca.* Liberibacter asiaticus’ genome. As an “accessory” genome component [14], *pSymA* may have originated from an ancestral plasmid and may have maintained individual and some small blocks of genes originally from host chromosomes. It has long been known that the *pSymA* megaplasmid of *S. meliloti*, in contrast to the circular chromosome or the other *S. meliloti* megaplasmid *pSymB*, specializes in carrying genes essential for establishing and maintaining intimate intracellular plant interactions with alfalfa. The megaplasmid *pSymA* itself is self-transmissible and upon transfer confers nodulation (*nod)*, nitrogen fixation (*nif*) and other symbiotic abilities in related bacteria with a similar genetic background or complement of chromosomal genes [15]–[17]. The chromosome of the tumor-inducing bacterial phytopathogen *Agrobacterium tumefaciens* encodes characteristics practically identical to those of *Rhizobium* microsymbionts, and members of the species also maintain pTi as an accessory genomic component. When bacterial cells recruit or accept transfer of the Ti plasmid or pSymA, either tumor-inducing pathogenicity or nitrogen-fixing symbiotic ability, respectively, is conferred on the host bacterium. *Agrobacterium* and *Rhizobium* were combined into a single genus within the Rhizobiaceae largely for this reason and to reject taxonomy based largely on plant interactions determined by extrachromosomal elements, i.e., to avoid plasmid-based nomenclature [4].

As *S. meliloti* is a close phylogenetic relative of ‘*Ca*. Liberibacter asiaticus,’ it is likely that the two bacteria deploy a similar repertoire of mechanisms for avoiding defenses elicited in host plant cells by their invasion, or, in the case of beneficial root nodule bacteria, recruitment or “welcomed entry” [18]. The goal of the present study was to compare predicted gene products encoded by the genome of ‘*Ca*. Liberibacter asiaticus’ with those encoded by genes carried on the megaplasmid *pSymA* of *S. meliloti* strain 1021. The rationale for identifying protein products of ‘*Ca*. Liberibacter asiaticus’ genes orthologous to those encoded by genes on *pSymA* is simply that such genes and their protein products may play direct or indirect role(s) in establishing and maintaining an intimate intracellular interaction with eukaryotes. We have identified a number of such interesting protein products.

## Results

### Maintenance of Homeostasis

In general, proteins encoded by *pSymA* genes are more similar in sequence alignment to those encoded by *S. meliloti* chromosomal orthologs than to orthologous proteins encoded by genes carried on the ‘*Ca.* Liberibacter asiaticus’ genome. There are two very notable exceptions where orthologs are shared only by pSymA and ‘*Ca.* Liberibacter asiaticus’ and not with the chromosome of *S. meliloti*. YP_003064907, the predicted protein product of a ‘*Ca*. Liberibacter asiaticus’ gene, is orthologous to NP_435608.2 (E = 5e-71) encoded by *pSymA* yet lacks an orthologous protein encoded by the chromosome of *S. meliloti* (Table 1). SMART analysis predicts that YP_003064907, has a cation efflux domain (E = 1.3e-31) from residues 15 to 313 of the 313 amino acid protein. This predicted protein also has BLAST matches with cobalt, zinc and cadmium efflux proteins (COG3965) [19]. *pSymA*-encoded NP_436297.2 is orthologous to YP_003064775 (E = 3e-30) encoded on the chromosome of ‘*Ca*. Liberibacter asiaticus’, but neither has an ortholog encoded by the *S. meliloti* chromosome (Table 1). SMART analysis of ‘*Ca*. Liberibacter asiaticus’ YP_003064775, annotated as a *nha*A gene, predicts a sodium/proton antiporter domain (E = 2.1e-113) at residues 4 to 379 of the 381 amino acid protein [20], [21]. These two protein pairs are the only two protein pairs uniquely shared by *pSymA* and ‘*Ca*. Liberibacter asiaticus’. A third orthologous protein pair comprised of NP_436118.1 (pSymA) and YP_003065337 (‘*Ca*. Liberibacter asiaticus’) is much more similar in amino acid sequence (E = 1e-134) than is NP_436118.1 and its ortholog encoded by the *S. meliloti* chromosome, NP_384912 (E = 1e-22). Bioinformatic analyses revealed that YP_003065337 is a potassium efflux protein. Control of intracellular pH is ascribed in its domain architecture. SMART analysis of YP_003065337 confidently predicts two domains as follows: an amino-terminal sodium/proton exchanger domain (E = 2.7e-72) from residues 4 to 394 and a non-overlapping TrkA domain (E = 8.3e-25) for redox regulation, from amino acids 451–566 of the 609 amino acid protein. ‘*Ca*. Liberibacter asiaticus’ YP_003065245, a potassium uptake protein, has E = 0 alignments with both *pSymA* (NP_436237.1) and *S. meliloti* chromosomal orthologs (Table 1). These proteins likely contribute to the maintainence of intracellular homeostasis through the regulation of pH and the control of mono- and divalent cation concentrations.

**Table 1:** Proteins shared between the *Sinorhizobium meliloti* plasmid pSymA, and the ‘*Ca*. Liberibacter asiaticus’ and *Sinorhizobium meliloti* chromosomes and with roles in the maintenance of homeostasis or signal transduction.

pSymA	*L. asiaticus*	E	AA	%	*S. meliloti*	E	Annotation
pSymA vs L. asiaticus					pSymA vs *S. meliloti*		
**Maintenance of Homeostasis**							
NP_435608.2	YP_003064907	5E-71	306	65	********	NA	cation diffusion facilitator transporter
NP_436297.2	YP_003064775	3E-30	333	44	********	NA	Na+/H+ antiporter
NP_436118.1	YP_003065337	1E-134	605	62	NP_384912	1E-22	Potassium efflux protein
NP_436237.1	YP_003065245	0E+00	624	67	NP_384938	0E+00	put potassium uptake protein
**Signal Transduction**							
NP_436498.1	YP_003064826	3E-16	239	49	NP_384498	6E-22	signal transduction histidine kinase
NP_436497.1	YP_003065306	1E-22	220	47	NP_385120	6E-35	2-component response regulator
NP_435549.2	YP_003065316	1E-12	225	46	NP_386067	2E-22	2-component sensor histidine kinase
NP_435864.1	YP_003064863	3E-49	394	53	NP_385908	1E-53	2-component sensor histidine kinase
NP_435916.1	YP_003064863	2E-16	268	43	NP_385564	4E-19	2-component sensor histidine kinase

Genbank numbers, E-values, number of amino acids (AA) in the homologous region of the protein and the percentage of similar amino acids (%) in pair wise comparisons are provided. The annotations are for the proteins in ‘*Ca*. Liberibacter asiaticus’.

### Signal Transduction

There are several orthologous proteins encoded by genes shared between *pSymA* and the ‘*Ca*. Liberibacter asiaticus’ and *S. meliloti* chromosomes that are predicted to function in sensing and signal transduction. For example, YP_003064826, a PAS/PAC sensor signal transduction kinase has a *pSymA* ortholog and a *S. meliloti* chromosomal ortholog. Several two-component response regulators or sensor histidine kinases are encoded both by genes on *pSymA* and the genome ‘*Ca*. Liberibacter asiaticus’, and include YP_003065306, which has a characteristic N-terminal CheY-like sensor and Trans_reg_C which are DNA-binding transcriptional activator/effector domains. YP_003064863, a two component sensor histidine kinase/response regulator hybrid protein, is a FixL-like oxygen-controlled histidine kinase homologous to several *pSymA*-encoded proteins (E = 3e-49 for the most similar ortholog) (Table 1).

### ABC-cassette Type Amino Acid Transporters

**Figure 1:**
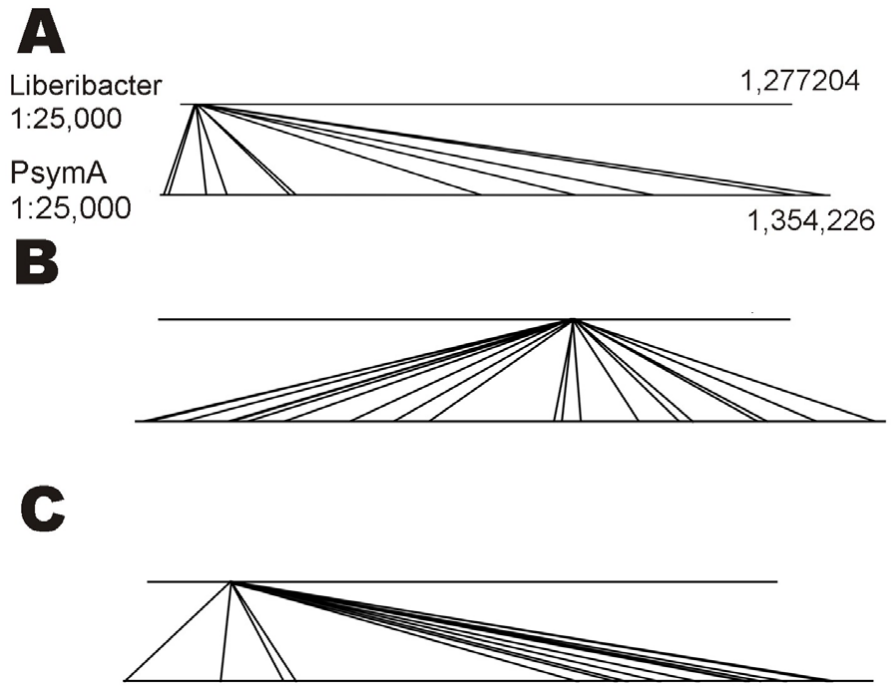
Distribution of genes on the *S. meliloti* pSymA megaplasmid that encode proteins orthologous to YP_003064586, annotated as an ABC-Type amino acid transporter in ‘*Ca*. Liberibacter asiaticus’ (A), that encode proteins orthologous to YP_003064948, annotated as a 3-ketoacyl-(acyl carrier protein) reductase in ‘*Ca*. Liberibacter asiaticus’ (B) and that encode proteins orthologous to YP_003064695, annotated as a LysR type regulator of transcription in ‘*Ca*. Liberibacter asiaticus’ (C).

ATP Binding Cassette type amino acid transporters comprise the largest class of genes shared by ‘*Ca*. Liberibacter asiaticus’, pSymA and the *S. meliloti* chromosome (Table 2). For most ABC-type transporter proteins encoded by ‘*Ca*. Liberibacter asiaticus’ genes there are multiple homologs on *pSymA,* dispersed over the megaplasmid (Table S1). YP_003064586 is an example of an ABC-type transporter protein from ‘*Ca*. Liberibacter asiaticus’. In addition to NP_435288.1 there are 10 more *pSymA*-encoded proteins homologous to YP_003064586 (E = 4e-67 up to 1e-14), and all are annotated as general amino acid transporters with an ATP binding site and other characteristics of an ABC type amino acid transporter. The corresponding genes are dispersed across the pSymA (Fig. 1A; Table 2).

**Table 2:** Proteins shared between the *Sinorhizobium meliloti* plasmid pSymA, and the ‘*Ca*. Liberibacter asiaticus’ and *Sinorhizobium meliloti* chromosomes with roles in ABC-Type transport and the synthesis of amino acids and metabolic cofactors.

pSymA	*L. asiaticus*	E	AA	%	*S. meliloti*	E	Annotation
pSymA vs L. asiaticus				pSymA vs *S. meliloti*			
**ABC-Type Transport Systems**							
NP_435288.1	YP_003064586	6E-87	257	76	NP_385581	1E-100	ABC transport
NP_436046.1	YP_003064753	9E-41	230	59	NP_386072	1E-50	proline/glycinebetaine ABC transporter
NP_436047.1	YP_003064754	3E-12	138	57	NP_385141	3E-19	proline/glycinebetaine ABC transporter
NP_435405.1	YP_003065117	2E-24	243	51	NP_387074	7E-65	ABC transporter, nucleotide binding
NP_435492.2	YP_003064552	2E-24	238	53	NP_387225	1E-92	put ATP binding ABC transporter
NP_436433.1	YP_003065525	3E-12	243	42	NP_387380	8E-21	put amino acid-binding periplasmic ABC
NP_435285.1	YP_003065526	7E-24	202	59	NP_387382	2E-27	amino acid ABC transporter permease
NP_435480.1	YP_003064636	1E-12	275	41	NP_384592	3E-17	ABC transporter permease
NP_435301.1	YP_003064972	5E-22	204	53	NP_386055	1E-66	thiamine transporter ATP-binding subunit
	**Synthesis of Amino Acids and Metabolic Cofactors**						
NP_436267.1	YP_003064901	5E-35	418	46	NP_386510	2E-70	adenosylmethionine-8-amino-7- transam
NP_436409.1	YP_003064762	1E-150	417	74	NP_385314	1E-161	Serine hydroxymethyl transferase
NP_436412.1	YP_003065324	8E-72	278	66	NP_384204	2E-81	Formyl tetrahydrofolate deformylase
NP_435712.1	YP_003064946	4E-63	422	53	NP_385250	6E-67	3-oxoacyl-(acylcarrierprotein) synthase II
NP_435713.1	YP_003064948	8E-78	247	70	NP_385248	1E-110	3-ketoacyl-(acyl-carrier-protein) reductase
NP_435500.1	YP_003064817	6E-13	232	43	NP_386931	2E-46	inositol monophosphatase family
NP_435501.1	YP_003064878	3E-75	436	56	NP_385027	8E-76	threonine synthase
NP_436504.1	YP_003064845	3E-33	427	43	NP_386695	4E-41	Acetyl ornithine transaminase
NP_435614.1	YP_003064846	5E-47	310	53	NP_384624	1E-47	ornithine carbamoyl transferase
NP_436258.1	YP_003065195	9E-13	218	43	NP_384531	4E-11	succinyl-diaminopimelate desuccinylase

Genbank numbers, E-values, number of amino acids (AA) in the homologous region of the protein and the percentage of similar amino acids (%) in pair wise comparisons are provided. The annotations are for the proteins in ‘*Ca*. Liberibacter asiaticus’.

YP_003064753 is a proline/glycine betaine ABC transporter protein with eight *pSymA*-encoded homologs (Table S1). YP_003064754, another proline/glycine betaine permease/ABC transporter permease protein, has two *pSymA*-encoded homologs. ‘*Ca*. Liberibacter asiaticus’ YP_003065117 and YP_003064552, both ATP-binding ABC-type transporter proteins, each have four homologs encoded by genes on *pSymA*. YP_003065525, a putative amino acid-binding periplasmic protein, is homologous to two *pSymA*-encoded predicted proteins. YP_003065526, an ABC-type amino acid transporter permease, has 7 homologs encoded by *pSymA* genes. These are examples of multiple, redundant shared homologs of ABC-type amino acid transport systems encoded by *pSymA*. ‘*Ca*. Liberibacter asiaticus’ YP_003064636 is an exception since this putative transmembrane ABC-type transporter permease has only one *pSymA*-encoded homolog, NP_435480.1 (Table 2). The multiplicity of ABC-type transporter homologs on *pSymA* presumably provides a repertoire of ABC transporters for *S. meliloti*, and is consistent with a high magnitude of importance for ABC transporter systems in *S. meliloti*. In contrast, ‘*Ca*. Liberibacter asiaticus’ lacks the general overall redundancy of ABC-type transporters present in *S. meliloti*.

Metabolic cofactors are also likely to be imported by ‘*Ca*. Liberibacter asiaticus’ and *S. meliloti* from the plant host. For example, essential vitamins are up taken by ABC-type transporters encoded by *pSymA* and the ‘*Ca*. Liberibacter asiaticus’ chromosome. YP_003064972, an ATP-binding component of an ABC transport complex for thiamine, has two homologs on both *pSymA* and the *S. meliloti* chromosome.

### Synthesis of Metabolic Cofactors and Amino Acids

In addition to transporter proteins, there are a large number of genes shared between ‘*Ca*. Liberibacter asiaticus’, *S. meliloti* and *pSymA* that encode proteins involved in the synthesis of metabolic cofactors (Table 2). ‘*Ca*. Liberibacter asiaticus’ YP_003064901 encodes adenosylmethionine-8-amino-7-oxononanoate transaminase (E = 5e-35). The enzyme 8-amino-7-oxononanoate synthase (AONS), which is pyridoxal 5′-phosphate-dependent, catalyzes 8-amino-7-oxononanoate synthesis, needed for biosynthesis of biotin. ‘*Ca*. Liberibacter asiaticus’ YP_003064762 is a serine hydroxymethyltransferase (SHMT) that catalyzes production of L-serine and tetrahydrofolate with an exceptionally low E-value when compared with its pSymA ortholog NP_436409. YP_003065324 encodes formyltetrahydrofolate deformylase, active in glyoxylate and dicarboxylate metabolism. YP_003064946 is a 3-oxoacyl-(acyl carrier protein) synthase, presumably involved in fatty acid synthesis, orthologous to NodE, a *pSymA*-encoded protein NP_435712.1 (E = 4e-63). YP_003064948, annotated as a 3-ketoacyl-(acyl carrier protein) reductase, is a dehydrogenase involved in fatty acid biosynthesis (COG1028). Megaplasmid pSymA encodes 20 proteins similar to YP_003064948 (E = 8e-78 to E = 2e-11) (Fig. 1B; Table S1).

Genes on pSymA with orthologs on the ‘*Ca*. Liberibacter asiaticus’ and *S. meliloti* chromosomes also encode enzymes involved in the synthesis of amino acids (Table 2). The predicted protein products of ‘*Ca*. Liberibacter asiaticus’ YP_003064878 and pSymA NP_435501.1 (E = 3e-75) have domains expected of threonine synthase, ThrC, a member of the TrpB tryptophan synthase superfamily. YP_003064845 is an acetylornithine transaminase protein, part of the arginine metabolism and lysine biosynthesis pathways. ‘*Ca*. Liberibacter asiaticus’ YP_003064846 and its orthologs in S. *meliloti*, encoded by genes on *pSymA* and its chromosome, are predicted to be an ornithine carbamoyl transferase, involved in arginine metabolism. YP_003065195 and its *S. meliloti* homolog are succinyl diaminopimelate desuccinylase involved in lysine biosynthesis. However, NP_436258.1, a similar protein encoded by pSymA, is annotated as an acetylornithine deacetylase, an enzyme which catalyzes a step in arginine metabolism. Plant hosts of ‘*Ca*. Liberibacter asiaticus’ are presumed to supply some amino acids to the obligate phytopathogen since certain genes for the biosynthesis of several amino acids were not found in the comparison of the chromosomes of ‘*Ca*. Liberibacter asiaticus’ and *S. meliloti* [2].

### Export of Bioactive Molecules

In addition to the proteins involved in the import or potential export of amino acids and metabolic cofactors, ‘*Ca*. Liberibacter asiaticus’ shares genes with pSymA and the *S. meliloti* chromosomes for several proteins used to export small bioactive molecules (Table 3). These include YP_003064686 an ABC-type transporter with excellent Blast alignments with chromosomal virulence gene (*chvD*) in *Agrobacterium*, *Bartonella and Brucella* [22].

**Table 3:** Proteins shared between the *Sinorhizobium meliloti* plasmid pSymA, and the ‘*Ca*. Liberibacter asiaticus’ and *Sinorhizobium meliloti* chromosomes with roles in the export of bioactive molecules, as chaperonins and in the regulation of transcription and translation.

pSymA	*L. asiaticus*	E	AA	%	*S. meliloti*	E	Annotation
pSymA vs L. asiaticus				pSymA vs S. meliloti			
	**Export of Bioactive Molecules**						
NP_435263.1	YP_003064686	9E-62	505	51	NP_385265	2E-87	put ABC transporter ATP-binding
NP_435718.1	YP_003065157	4E-30	239	51	NP_385995	1E-35	ABC transporter nucleotide binding
NP_435856.2	YP_003065113	5E-33	241	51	NP_385127	2E-45	Serine protease DO like protease
	**Chaperonins**						
NP_435305.2	YP_003065051	2E-15	175	47	NP_385003	7E-27	chaperone protein DnaJ
NP_435342.1	YP_003065326	8E-13	70	67	NP_386217	1E-18	Cold shock protein
NP_435641.1	YP_003065262	0E+00	526	77	NP_384898	0E+00	Chaperonin GroEL
NP_435311.1	YP_003065261	4E-22	95	71	NP_384899	8E-39	co-chaperonin GroES
	**Regulation of Transcription and Translation**						
NP_436119.2	YP_003064695	2E-20	253	48	NP_384882	1E-43	put Transcriptional regulator
NP_435689.2	YP_003065329	2E-33	245	51	NP_386901	8E-60	put σ-54-dependent trans reg
NP_436184.1	YP_003065196	7E-32	132	66	NP_385105	1E-35	transcriptional regulator
NP_436495.1	YP_003065365	7E-49	667	44	NP_386250	3E-58	DNA helicase II
NP_436130.1	YP_003065000	2E-15	311	45	NP_387206	8E-28	site-specific recombinase XerD
NP_435253.1	YP_003064676	7E-19	135	54	NP_385460	1E-16	translation elongation factor Tu
NP_436002.1	YP_003065391	7E-20	311	41	NP_385418	1E-34	asp/glut-tRNA amidotransferase

Genbank numbers, E-values, number of amino acids (AA) in the homologous region of the protein and the percentage of similar amino acids (%) in pair wise comparisons are provided. The annotations are for the proteins in ‘*Ca*. Liberibacter asiaticus’.

The ‘*Ca*. Liberibacter asiaticus’ genome encodes two *nod*I homologs, likely involved in exporting lipid virulence factors. YP_003065157, an ATP-binding ABC-type transporter protein with a yhbG domain, is similar to *pSymA* nodI, a multidrug efflux-like protein that secretes lipids and lipopolysaccarides including cell wall and outer membrane or Nod factor components. Interestingly, pSymA encodes six homologs of YP_003065157, consistent with important roles for these proteins in the *S. meliloti*/alfalfa interaction. When ABC-type transporters of ‘*Ca.* Liberibacter asiaticus’ were blasted against the Transporter Classification Database (http://www.tcdb.org/), YP_003065330, annotated as an ABC-type transporter nucleotide binding/ATPase, was homologous with Q2G2M9 (4.00E-088), a putative multi-drug export ATP-binding/permease protein of *Staphylococcus aureus,*SAOUHSC_02003. Like YP_003065157, YP_003065330 aligns best with nodI NP_435718.1 (2.00E-021) encoded by *pSymA*.

‘*Ca*. Liberibacter asiaticus’ YP_003065113, orthologous to pSymA-encoded protein NP_435856.2 (E = 5e-33), a serine protease DO protease, has a PDZ-metalloprotease domain, a PDZ serine protease domain and a trypsin domain, all fused into a single multi-domain protease product. This protein, a probable virulence factor, is likely to protrude from ‘*Ca*. Liberibacter asiaticus’ into the host cell where it can presumably digest host proteins using zinc as a cofactor.

### Chaperonins

Several chaperonin proteins are encoded by orthologous genes shared by ‘*Ca*. Liberibacter asiaticus’, pSymA and *S. meliloti* (Table 3). YP_003065051 is a DnaJ molecular chaperone protein with one orthologous protein encoded by pSymA, NP435305.2. Cold shock protein YP_003065326 has three similar proteins encoded by pSymA. YP_003065262 (GroEL) is orthologous with both pSymA NP_435641.1 (E = 0) and with pSymA NP_435310.1 (E = 1e-166). Co-chaperonin GroES YP_003065261 is orthologous with pSymA-encoded NP_535311.1 (E = 4e-22) and NP_435642.1 (E = 9e-21).

### Regulation of Transcription and Translation

‘*Ca*. Liberibacter asiaticus’, pSymA and *S. meliloti* share several proteins that regulate transcription (Table 3). YP_003064695 is a member of the LysR family of transcription regulator proteins. Its importance for plant interaction is indicated by at least 14 LysR-like proteins encoded by and distributed across pSymA (E = 1e-21 to E = 1e-10) (Fig. 1C; Table S1). Other genes encoding transcriptional regulators are not as extensively duplicated on pSymA, consistent with LysR family transcription regulators being of unique importance in plant interaction in *S. meliloti* [23]. The ‘*Ca*. Liberibacter asiaticus’ genome, by contrast, encodes only YP_003064695, annotated as transcriptional regulator with a LysR region. YP_003065329, a putative sigma-54-dependent transcription regulator protein is similar to NP_435689 in pSymA annotated as NifA. NifA is a DNA-binding ATPase and CheY-like two-component sensor-based response regulator that is known to activate expression of genes involved in nitrogen metabolism. YP_003065196, encodes a transcription-activating MucR-like protein [24], with two orthologs on pSymA. MucR proteins regulate production of extracellular polysaccharides which could contribute to biofilm formation in phloem cells of citrus inhabited by ‘*Ca.* Liberibacter asiaticus.’ YP_003065365 and pSymA NP_436495 (E = 7e-49) are encoded by orthologous genes for a UvrD2 DNA helicase II, or superfamily I DNA and RNA helicase. ‘*Ca*. Liberibacter asiaticus’ YP_003065000 and pSymA orthologs NP_436130.1 and NP_436426.1 encodes a XerD recombinase/integrase. YP_003064676, a Tu translation elongation factor, is orthologous to pSymA-encoded NP_435253 (E = 7e-19) and to NP_435715.1 (E = 6e-18), and is involved in the synthesis of proteins. YP_003065391 encodes aspartyl/glutamyl-tRNA amidotransferase, an enzyme known to allow formation of correctly charged asparagine or glutamine tRNAs in organisms which lack either or both a asparaginyl-tRNA synthetases or a glutaminyl-tRNA synthetase.

### Cell Surface

‘*Ca.* Liberibacter asiaticus’ and pSymA share several genes encoding proteins that contribute to a functional architecture of the cell surface (Table 4). A UDP-glucose-4-epimerase, YP_003064741 probably has a role interconverting monosaccarides needed for exopolysaccaride synthesis. Cyclic diguanylate controls biofilm formation [25]. YP_003064881, a putative prokaryotic sensory diguanylate cyclase/phosphodiesterase protein, has two orthologs on pSymA, NP_435318.2 (E = 1e-42) and NP_436089.2 (E = 7e-42). YP_003065366 encodes an OmpA/MotB-like protein similar with *S. meliloti* pSymA NP_435574.1 (E = 8e-14) and the *S. meliloti* chromosome NP_384380 (E = 2e-16). Pilus assembly gene clusters on pSymA have orthologs on the ‘*Ca*. Liberibacter asiaticus’ chromosome. Proteins YP_003065134 (NP_436096, E = 5e-50), YP_003065135 (NP_436097, E = 3e-49), YP_003065137 (NP_436098, E = 1e-178; NP_435955, E = 2e-35), YP_003065140 (NP_435332.2, E = 9e-45) and YP_003065141 (NP_436102.1, E = 3e-29) are all components of a pilus assembly for ‘*Ca*. Liberibacter asiaticus’. YP_003065138, a pilus-associated response regulator receiver protein, likely regulates production of pili based on signals received from the environment.

**Table 4:** Proteins shared between the *Sinorhizobium meliloti* plasmid pSymA, and the ‘*Ca*. Liberibacter asiaticus’ and *Sinorhizobium meliloti* chromosomes with roles in the modification of cell surface structures.

pSymA	*L. asiaticus*	E	A A	%	*S. meliloti*	E	Annotation
pSymA vs L. asiaticus				pSymA vs S. meliloti			
**Cell Surface Structure**							
NP_435626.1	YP_003064741	4E-11	188	46	NP_384213	7E-18	UDP-glucose-4-epimerase
NP_436089.2	YP_003064881	7E-42	451	49	NP_385065	2E-95	sensory box/GGDEF family
NP_435318.2	YP_003064881	1E-42	426	49	NP_385065	3E-76	sensory box/GGDEF family
NP_435574.1	YP_003065366	8E-14	96	59	NP_384380	2E-16	OmpA/MotB/outer membrane
NP_436096.2	YP_003065134	5E-50	254	57	NP_384252	1E-62	Pilus component
NP_436097.1	YP_003065135	3E-49	179	72	NP_384251	7E-68	Pilus component
NP_436098.1	YP_003065137	1E-178	408	83	NP_384250	0E+00	component of type IV pilus
NP_435332.2	YP_003065140	9E-45	455	49	NP_384247	2E-43	put pilus assembly
NP_436102.1	YP_003065141	3E-29	200	55	NP_384246	2E-32	put pilus assembly
NP_436101.1	YP_003065138	1E-66	397	55	NP_384249	7E-76	Response regulator receiver (pilus)
NP_436359.1	YP_003064721	3E-40	204	57	NP_387423	1E-64	lytic murein transglycosylase
NP_436481.1	YP_003065165	0E+00	711	70	NP_385436	0E+00	Peptidoglycan synthetase
NP_436482.1	YP_003065516	1E-93	416	60	NP_386288	1E-100	penicillin-binding transmembrane
NP_435728.1	YP_003065356	0E+00	608	76	NP_435728	0E+00	glucosamine-6-Phos transporter

Genbank numbers, E-values, number of amino acids (AA) in the homologous region of the protein and the percentage of similar amino acids (%) in pair wise comparisons are provided. The annotations are for the proteins in ‘*Ca*. Liberibacter asiaticus’.

A transglycosylase, YP_003064721 in ‘*Ca*. Liberibacter’ asiaticus’ and pSymA-encoded NP_436359.1 (E = 3e-40), could produce cell wall degradation products. YP_003064721 signal peptide protein orthologous to pSymA NP_436359.1 and is a putative membrane-bound lytic murein transglycosylase (E = 3e-40).

YP_003065165, a penicillin-binding peptidoglycan synthase, is orthologous to NP_ 436481.1 (E = 0). A transmembrane protein that also binds penicillin, YP_003065516 is orthologous to pSymA NP_436482.1 (E = 1e-93). ‘*Ca*. Liberibacter asiaticus’ YP_003065356 (Table 1), a glucosamine-fructose-5-phosphate aminotransferase, needed for N-acetyl D-glucosamine synthesis, aligns with pSymA-encoded NP_435728.1 (E = 0).

### Electron Transfer

**Figure 2:**
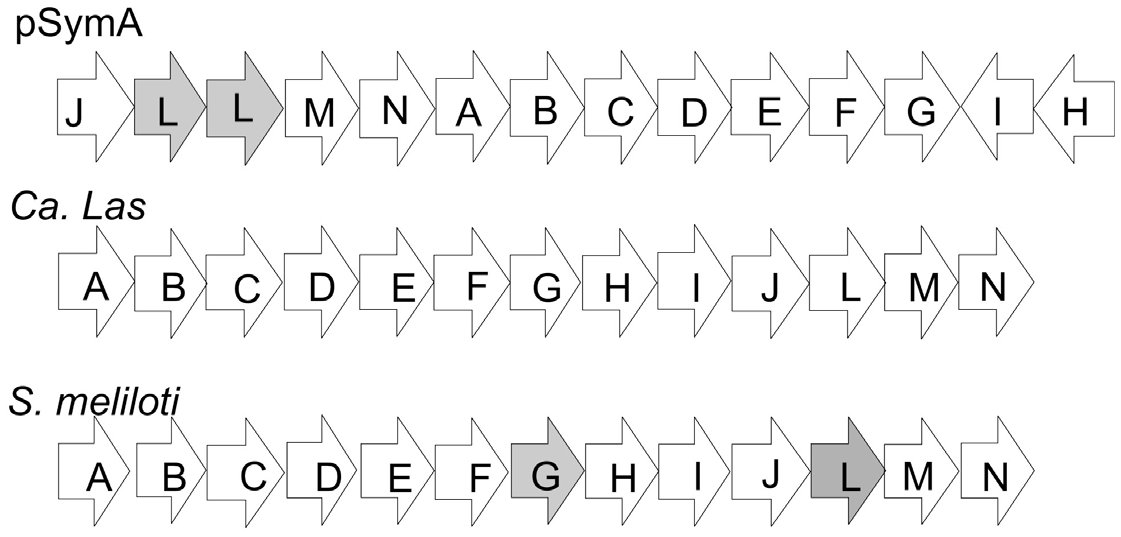
Diagram showing the organization of the genes encoding the components of the NADH dehydrogenase complex on pSymA, ‘*Ca*. Liberibacter asiaticus’ and *S. meliloti*.

An important conserved microsyntenous orthologous gene (MOG) cluster, occurs in the ‘*Ca*. Liberibacter asiaticus’ genome and in the genomes of four other members of the Rhizobiales that were analyzed previously [26]. This conserved MOG encodes thirteen subunits that comprise a “mitochondrial-like” Type I respiratory complex (Table 5). Genes encoding this respiratory complex are also found on pSymA, and orthologous proteins specified by ‘*Ca*. Liberibacter asiaticus’ chromosomal genes include the following: Chain A, NuoA2 NADH: ubiquinone oxidoreductase YP_003065265 and NP_436080.1 (E = 9e-14); Chain B, or NuoB2 YP_003065266 and NP_436079 (E = 6e-48); Chain C, or NuoC2 YP_003065267 and NP_436078.1 (E = 6e-33); Chain D, or NuoD2 YP_003065268 and NP_436077.1 (E = 1e-110); Chain E or NuoE2, YP_003065269 and NP_436076.1 (E = 4e-24) and Chain F or NuoF2 YP_003065271 and NP_436075.1 (E = 1e-104). Chains G, H and I/J or NuoG2, NuoH2, a multi-subunit ubiquinone oxidase have the following orthologs: YP_003065272 and NP_436074.1 (E = 2e-62); YP_003065273 and NP_436071.1 (E = 1e-51), YP_003065274 and NP_436072.1 (E = 1e-22), and YP_003065275 and NP_436087.2 (E = 8e-11). YP_003065278 is orthologous with pSym proteins NP_436085.1 and NP_436083.4 annotated as, respectively, a multidomain K/L oxidoreductase, a NADH ubiquinone oxidase (COG1009), or a multi-domain Na+/K+ antiporter, largely synonomous, involved in energy generation, cation efflux or intracellular pH control. ‘*Ca*. Liberibacter asiaticus’ YP_003065279 and pSymA NP_436082.1 (E = 2e- 92) are chain M of ubiquinone oxidoreductase. ‘*Ca*. Liberibacter asiaticus’ YP_003065280 and pSymA NP_436081.1 are chain N (E = 4e-57). Nearly all orthologous proteins shared between pSymA and *S. meliloti* chromsome have lower E-values aligned with each other than do orthologs shared between pSymA and the ‘*Ca*. Liberibacter asiaticus’ chromosome (Tables 1–4). However the amino acid sequence similarities between protein components of the respiratory complex I encoded by pSymA, and the ‘*Ca.* Liberibacter asiaticus’ genome and the *S. meliloti* chromosome are highly comparable with each other (Table 5). The organization of the genes in genomic regions encoding the NADH dehydrogenase complex is the same for *S. meliloti* and ‘*Ca*. Liberibacter asiaticus’ (Fig. 2). NuoG and NuoL are encoded by genes in the expected cluster on the *S. meliloti* chromosome. But the e-values obtained by pBlast of pSymA NuoG and NuoL vs *S. meliloti* are better for genes found elsewhere on the *S. meliloti* chromosome. The best match for pSymA NuoG (NP_43074) on the *S. meliloti* chromosome is a NAD-dependent formate dehydrogenase alpha subunit (NP_387115; E = 2e^−95^). The NADH dehydrogenase subunit G (NP_385378; E = 8e^−77^) is the second best result. This protein is transcribed and translated from a gene with the expected position in the NADH dehydrogenase MOG and aligns well with the ‘*Ca*. Liberibacter asiaticus’ NuoG (e = 0). A similar finding was obtained when pSymA NP_436085 encoding the NuoL subunit was used in a blast search of the chromosome of *S. meliloti*. The best matches were to proteins annotated as monovalent cation/H+ antiporter subunits located at different positions on the chromosome. NADH dehydrogenase subunit L (NP_385383; E = 7e^−25^) encoded by the chromosome was significantly less well-aligned. As was seen for NuoG, this protein is transcribed from a gene with the expected position in the NADH dehydrogenase MOG and aligns well with the ‘*Ca*. Liberibacter asiaticus’ NuoL (e = 0). Thus these proteins likely function in the role of NuoL. The NADH dehydrogenase gene cluster on pSymA has a different arrangement of genes (Fig. 2). Genes encoding NuoA-NuoG in the center of the Nuo gene cluster are flanked on one side by genes encoding NuoJ – NuoN and by genes, transcribed from the opposite strand, that encode NuoH and NuoI on the other side.

**Table 5:** Proteins shared between the *Sinorhizobium meliloti* plasmid pSymA, and the ‘*Ca*. Liberibacter asiaticus’ and *Sinorhizobium meliloti* chromosomes with roles in electron transfer.

pSymA	*L. asiaticus*		E	A A	%	*S. meliloti*	E	Annotation
pSymA vs L. asiaticus					pSymA vs S. meliloti			
NP_436080.1		YP_003065265	9E-14	109	53	NP_385370	3E-14	NADH dehydrogenase subunit A
NP_436079.1		YP_003065266	6E-48	141	79	NP_385371	2E-47	NADH dehydrogenase subunit B
NP_436078.1		YP_003065267	6E-33	142	60	NP_385372	1E-29	NADH dehydrogenase subunit C
NP_436077.1		YP_003065268	1E-110	393	64	NP_385373	1E-106	NADH dehydrogenase subunit D
NP_436076.1		YP_003065269	4E-24	169	53	NP_385375	3E-22	NADH-quinone oxidoreductase subunit E
NP_436075.1		YP_003065271	1E-104	420	64	NP_385376	1E-105	NADH dehydrogenase I subunit F
NP_436074.1		YP_003065272	2E-62	410	54	NP_385378*	2E-65	NADH dehydrogenase subunit G
NP_436071.1		YP_003065273	1E-51	311	55	NP_385379	2E-54	NADH dehydrogenase subunit H
NP_436072.1		YP_003065274	1E-22	136	54	NP_385380	4E-23	NADH dehydrogenase subunit I
NP_436087.2		YP_003065275	8E-11	54	78	NP_385381	6E-10	NADHdehydrogenasesubunit J
NP_436085.1		YP_003065278	2E-23	461	42	NP_385383*	1E-22	NADH dehydrogenase subunit L
NP_436083.4		YP_003065278	3E-20	180	52	NP_385383*	1E-19	NADH dehydrogenase subunit L
NP_436082.1		YP_003065279	2E-92	485	54	NP_385384	1E-100	NADH dehydrogenase subunit M
NP_436081.1		YP_003065280	4E-57	427	50	NP_385385	2E-75	NADH dehydrogenase subunit N
NP_436008.1		YP_003064781	3E-73	249	71	NP_436008	1E-128	electron transfer flavoprotein beta
NP_435693.1		YP_003064781	4E-14	186	52	NP_435693	8E-18	electrontransfer flavoprotein beta
NP_436007.1		YP_003064782	2E-77	316	69	NP_436007	1E-150	Electron transfer flavoprotein alpha
NP_435692.1		YP_003064782	2E-20	245	48	NP_386750	3E-21	Electron transfer flavoprotein alpha
NP_435691.1		YP_003065437	1E-18	326	43	NP_385139	6E-09	flavoprotein ubiquinone oxidoreductase
NP_435265.2		YP_003064703	2E-26	334	43	NP_385059	1E-32	quinone oxidoreductase
NP_435950.1		YP_003064703	4E-12	269	40	NP_386798	5E-42	quinoneoxidoreductase
NP_435375.1		YP_003065174	3E-76	425	56	NP_384973	7E-99	prob FAD-dependent oxidoreductase
NP_435937.1		YP_003065041	9E-20	187	51	NP_384479	3E-19	coproporphyrinogen III oxidase
NP_436285.1		YP_003064696	1E-22	134	61	NP_385673	1E-31	thioredoxin reductase (NADPH)

Genbank numbers, E-values, number of amino acids (AA) in the homologous region of the protein and the percentage of similar amino acids (%) in pair wise comparisons are provided. The annotations are for the proteins in ‘*Ca*. Liberibacter asiaticus’. *indicates proteins that were the second or third best match by Blast.

Other proteins are encoded by genes shared by ‘*Ca*. Liberibacter asiaticus’, pSymA and the chromosome of *S. meliloti*, and have roles in energy generation. YP_003065437, is a probable electron transfer flavoprotein-quinone oxidoreductase or FixC. YP_003064703 is orthologous with both *pSymA*-borne NP_435265.2 and NP_435950.1, which are zinc-dependent NADPH quinone oxidoreductases, COG0604, and are important in energy production. YP_003064781 and YP_003064782, encoded by adjacent genes, are orthologs of NP_436008.1 and NP_436007.1 (E = 3e-73 and 2e- 77 respectively). Both genes have two adjacent ortholog pairs on both pSymA and the *S. meliloti* chromosome. YP_003065174 and NP_435375.1 (E = 3e-76) are annotated as energy yielding FAD-dependent dehydrogenases. YP_003065041 is coproporphyrinogen III oxidase and is orthologous to a pSymA-borne locus NP_435937.1. The protein has two domains; N-terminal Radical-SAM super family and a C-terminal HemN_C superfamily, which obtains electrons from a flavodoxin-like system regenerated by a nicotinamide cofactor-dependent flavodoxin [27]. ‘*Ca*. Liberibacter asiaticus’ YP_003064696 is a NADPH thioredoxin reductase orthologous to NP_436285.1.

## Discussion

The premise for identifying protein products of ‘*Ca*. Liberibacter asiaticus’ orthologous to those encoded by genes on *pSymA* was an hypothesis that such genes and their protein products in all likelihood play direct or indirect role(s) in establishing and maintaining an intimate intracellular interaction with eukaryotes. ‘*Ca*. Liberibacter asiaticus’ has an obligatory requirement for a eukaryotic host, and there are multiple orthologs of these genes in *Sinorhizobium meliloti.* These factors preclude mutagenesis studies at the present time to test this hypothesis. In particular, we discovered that there are several orthologous genes in the ‘*Ca*. Liberibacter asiaticus’ genome and on *pSymA* that encode proteins with vital roles in maintaining intracellular homeostasis for mono- and divalent cations and pH. Genes encoding a Na^+^/H+ antiporter [20], [21] and a cation efflux antiporter protein that secretes excess metal ions Cd^++^, Zn^++^ or Co^++^ in exchange for K^+^ and H^+^ [19], [28] were identified as orthologs shared exclusively by *pSymA* and ‘*Ca*. Liberibacter asiaticus,’ i.e., not found on the *S. meliloti* chromosome. In a previous study we compared the proteins encoded by the circular chromosomes of ‘*Ca*. Liberibacter asiaticus’ and four other members of the Rhizobiales. In that study the cation efflux antiporter (YP_003064907) was found to be uniquely shared between ‘*Ca*. Liberibacter asiaticus’ and phytopathogenic *Agrobacterium tumefaciens* C58 (NP_354015; 3E-84). The Na^+^/H^+^ antiporter (YP_003064775) shared between pSymA and ‘*Ca*. Liberibacter asiaticus’ is also shared with 10 species or strains of the *Bartonellaceae* (6E-60–4E-48), but not with the other members of the Rhizobiales studied [26]. This suggests that this protein is used to facilitate an intracellular lifestyle. In addition, another protein encoded by genes shared by *pSymA* and the ‘*Ca*. Liberibacter asiaticus’ and *S. meliloti* chromosomes, is a glutathione-gated potassium efflux protein that exchanges potassium for sodium and protons and thereby modulates cytoplasmic pH [29], [30]. This orthologous protein pair encoded by both *pSymA* and the ‘*Ca*. Liberibacter asiaticus’ genome align with an e-value that is 110 orders of magnitude lower than the alignment of the orthologous proteins encoded by *pSymA* and the *S. meliloti* chromosome. Thus, the maintenance of intracellular pH and ion homeostasis are likely critical functions of proteins encoded by genes shared uniquely by ‘*Ca*. Liberibacter asiaticus’ and *pSymA* (but not the *S. meliloti* chromosome). Other ‘*Ca*. Liberibacter asiaticus’ genes with orthologs on *pSymA* likely encode proteins with either direct or indirect nutritional or energy roles in establishing and maintaining an intimate intracellular interaction with eukaryotic hosts.

We identified 86 ‘*Ca*. Liberibacter asiaticus’ genes with 182 probable orthologs on pSymA and the *S. meliloti* chromosome. Most of the orthologous proteins encoded by *pSymA* and the *S. meliloti* chromosome produced alignments with substantially more significant E-values than orthologous proteins encoded by ‘*Ca.* Liberibacter asiaticus’ and *pSymA*. Individual genes encoded by the reduced genome of ‘*Ca*. Liberibacter asiaticus’ may have many orthologs or paralogs carried by the *pSymA* megaplasmid besides additional similar genes on the chromosome of *S. meliloti*. An example is YP_003064948, a 3-ketoacyl-(acyl-carrier-protein) reductase with 20 similar proteins encoded by pSymA. Thus, numerous physiological and niche adaptations conferred upon *S. meliloti* by *pSymA* may not be available to ‘*Ca*. Liberibacter asiaticus.’ However, in a reduced genome such as that of ‘*Ca*. Liberibacter asiaticus’, genes that are retained may function with relaxed substrate specificity [31], [32], compensating for the lack of multiple homologs in the ‘*Ca*. Liberibacter asiaticus’ genome. The multiplicity of pSymA orthologs complicates any mutagenesis strategy to definitively establish their roles in *S. meliloti*.

Protein products not constitutively expressed may be considered as candidates for being involved with niche specialization or pathogenesis; *S. meliloti* has conditionally expressed genes that are especially important for nitrogen fixation and nodulation symbiosis. YP_003064695 is a putative regulator of transcription by ‘*Ca*. Liberibacter asiaticus’, with 14 related proteins in pSymA, many of which are annotated as LysR transcriptional regulatory proteins. Despite considerable conservation both structurally and functionally, LysR-type transcriptional regulators regulate a diverse set of genes, including those involved in virulence, metabolism, quorum sensing and motility [33], [34]. In *S. meliloti*, LysR proteins function in exquisite control of a sequence of events leading to root colonization, initiation of nodulation and subsequent differentiation of nodules containing *S. meliloti* [23]. Fatty acid products of the 3-ketoacyl-(acyl-carrier-protein) reductase orthologs are also used to complete the structure of nodulation factors. This may be why the extensive collection of LysR regulatory elements and 3-ketoacyl-(acyl-carrier-protein) reductases encoded on *pSymA* are not found on the ‘*Ca*. Liberibacter asiaticus’ chromosome. Thus, ‘*Ca*. Liberibacter asiaticus’ may have much less capability for finely nuanced control of the expression of genes, consistent with its obligatory intracellular lifestyle. Parasitism of citrus by ‘*Ca*. Liberibacter asiaticus’ follows direct injection of the pathogen into the host and results in systemic invasion of phloem tissues throughout the host [9], [10], and differentiated nodules are not produced.

Genes with orthologs on both pSymA and the chromosome of *S. meliloti* are largely specialized for symbiosis and nitrogen metabolism in *S. meliloti* and include 48 ABC-type transporter genes. The genome of ‘*Ca*. Liberibacter asiaticus’ encodes only 11 ABC-type transporter proteins that are orthologous to pSymA-encoded proteins, compared with a total of about 40 ABC-type transporters encoded by the chromosome [2]. These transporters likely serve to import sugars, amino acids and precursors and purines and pyrimidines into ‘*Ca*. Liberibacter asiaticus’ from the host. The genome of ‘*Ca*. Liberibacter asiaticus’ is known to be deficient in genes used in the biosynthetic pathways of these compounds [2], [12].

ABC-type transporters may also be used to export small molecules by ‘*Ca*. Liberibacter asiaticus’. YP_003064989 is an ABC-type transporter protein encoded by ‘*Ca*. Liberibacter asiaticus’ that likely functions to export glucans for osmoprotection. A class of proteins that confer multiple drug resistance, are called ‘RND’ for Resistance, Nodulation and Division [35]. RND-type proteins confer drug resistance based on rapid efflux of drugs from the bacterial cell. YP_003065157, is an ATP-binding ABC-type transporter protein with a yhbG domain, and is orthologous to *pSymA* NodI, a protein that is required for nodulation of alfalfa by *S. meliloti*. NodI-like proteins secrete lipids and lipopolysaccarides such as cell wall, outer membrane or Nod factor components needed to stimulate the differentiation of nodules by the host. Very interestingly pSymA encodes six homologs of YP_003065157. Orthologs of ABC-type transporters encoded by ‘*Ca*. Liberibacter asiaticus’ were identified with the “Transporter Classification Database” (http://www.tcdb.org/). YP_003065330, annotated as an ABC-type transporter with nucleotide binding and ATPase domains was homologous to Q2G2M9 (4.00E-88), annotated as a putative multi-drug export ATP-binding/permease protein, *Staphylococcus aureus* SAOUHSC_02003. YP_003065330 aligns best with nodI NP_435718.1 (2.00E-021) encoded by *pSymA*. Thus these proteins at the least share similar structural motifs.

Genes encoding type IV pili may have evolved from structures used for DNA transfer but it is now known that virulence proteins may be secreted by components of a type IV pili [36]. Genes encoding type IV pili are shared between ‘*Ca*. Liberibacter asiaticus’, *S. meliloti*, and pSymA. Effectors directly involved with the establishment of ‘*Ca.* Liberibacter asiaticus’ as an intracellular pathogen living within the phloem cells of citrus, may be transferred from the bacterium to its host by type IV secretion involving pilus assembly proteins. If pili are produced by ‘*Ca*. Liberibacter asiaticus’, they may also have roles in twitching motility and biofilm formation as in *Xylella fastidiosa* [37].

Why is the entire *S. meliloti* chromosomal respiratory complex I gene set duplicated on *pSymA*? Plants respond to bacterial pathogens by producing a sustained oxidative burst leading to apoptosis. The respiratory complex protein NuoG can also be used by *Mycobacterium tuberculosis* to pump protons out of the cell to neutralize such superoxide radicals [38]. This may explain the duplication of the genes encoding the respiratory complex on pSymA and provide an additional role for *Nuo*G in the ‘*Ca*. Liberibacter asiaticus’/citrus interaction. Homeostasis with respect to sodium is also of critical importance to both *S. meliloti* and ‘*Ca*. Liberibacter asiaticus’ as discussed above, and in addition to pumping protons out of the cell to create the proton motive force needed for the synthesis of ATP, respiratory complex I of at least some Gram negative bacteria can also transport sodium ions out of the cells [39]. Besides an obvious potential gene dosage effect, redundant copies carried on an extrachromosomal element provide a palette for gene evolution. However in spite of the potential for diversification of these genes afforded to pSymA, substantial diversification is not apparent since the E-values for the orthologs encoded by pSymA, ‘*Ca*. Liberibacter asiaticus’ and *S. meliloti* are very similar.

Most ‘*Ca*. Liberibacter asiaticus’ protein products that align significantly with proteins encoded by pSymA also align significantly with predicted protein products encoded by the *S. meliloti* chromosome. Thus, although present as single copies for the most part in ‘*Ca*. Liberibacter asiaticus’, these genes are present in multiple copies per replicon in the free-living microsymbiont. The presence of multiple orthologs in pSymA of ‘*Ca*. Liberibacter asiaticus’ proteins such as the amino acid transporter YP_003064586, the fatty acid dehydrogenase YP_003064948 and the LysR transcriptional regulator YP_003064695, suggests that their products are of particular importance in host/microbe interactions, and are likely to be examples of a single protein acquiring multiple functions in an organism with a reduced genome [32]. ‘*Ca*. Liberibacter asiaticus’ very notably also has two or three genes shared with pSymA but not the *S. meliloti* chromosome. These genes are predicted to produce proteins that generate a proton gradient with ATP-producing potential, maintain sodium and potassium homeostasis, prevent over accumulation of divalent metal cations and maintain intracellular pH. Thus the maintenance of intracellular homeostasis is potentially a vital requirement for successful colonization of citrus and psyllids by ‘*Ca*. Liberibacter asiaticus’. In an obligate pathogen, cellular vitality and pathogenesis are tied together.

## Materials and Methods

The genome of ‘*Ca*. Liberibacter asiaticus’ was compared with both the circular chromosome and megaplasmids, especially pSymA, of *S. meliloti*. To identify orthologous proteins, predicted amino acid sequences (‘*Ca.* Liberibacter asiaticus’ strain psy62, RefSeq NC_012985; *Sinorhizobium meliloti* strain 1021 RefSeq NC_003047, and pSymA RefSeq NC_003037) were downloaded from NCBI. Using default BLAST parameters, each predicted amino acid sequence from the ORFs identified on pSymA of the *S. meliloti* 1021 genome was BLASTed against the predicted amino acid sequences of the ORFs on the chromosomes of ‘*Ca*. Liberibacter asiaticus’ and *Sinorhizobium meliloti* strain 1021. Perl scripts and Excel spreadsheets were created to identify hits between genomes with low, negative e-values and to extract annotations from Genbank. In addition, amino acid similarity and length of the blast hit was extracted from each top hit from the BLAST output. First, a general analysis was done extracting predicted protein products having BLAST alignment values of e -10 or lower. Similar or homologous proteins were also identified manually where possible to be consistent with the annotations from the authors of each respective genome. That is, annotations of ORFs were extracted from NCBI annotations of the genome of ‘*Ca*. Liberibacter asiaticus’ and megaplasmid pSymA of *Sinorhizobium meliloti* strain 1021, respectively. Thus, the matching homolog might not have the best e-value or match with the top hit as many proteins share the same domain structure or amino acid similarity, but are functionally quite different. Similarly, for proteins of interest that were unique to each genome, proteins were sorted for positive e-values, signifying that an orthologous protein was not encoded in a respective genome. Orthologous genes from the ‘*Ca*. Liberibacter asiaticus chromosome, the *S. meliloti* chromosome and megaplasmid pSymA were mapped on linear representations of the respective genophores opened at their origins of replication.

## Supporting Information

Table S1 **Proteins shared between the Sinorhizobium meliloti plasmid pSymA, and the ‘Ca. Liberibacter asiaticus’ and Sinorhizobium meliloti chromosomes.** The protein IDs and annotations used by NCBI and the e-values of pairwise comparisons are provided.
(XLSX)
